# Editorial for Special Issue: Anthocyanin

**DOI:** 10.3390/molecules26092496

**Published:** 2021-04-25

**Authors:** Paula Silva, Luciana Mosca

**Affiliations:** 1 Laboratory of Histology and Embryology, Institute of Biomedical Sciences Abel Salazar (ICBAS), Rua de Jorge Viterbo Ferreira nº228, 4050-313 Porto, Portugal; 2 iNOVA Media Lab, ICNOVA, Universidade Nova de Lisboa, 1069-061 Lisboa, Portugal; 3 Department of Biochemical Sciences, Sapienza University, p.le Aldo Moro, 5, 00185 Rome, Italy

What do jabuticaba (*Myrciaria jaboticaba*), Jamun berry (*Syzygium cumini*), Malay apple (*Syzygium malaccense*), crimson glory vine (*Vitis coignetiae*) and roselle (*Hibiscus sabdariffa*) have in common? They are good sources of anthocyanins, and this Special Issue collects different studies showing their characteristics. Anthocyanins are widespread water-soluble pigments that have important roles in the propagation, protection, and physiology of higher plants. Anthocyanins are mainly present in flowers, cereals, and root vegetables, being the greatest in quantity in fruits, especially, grapes and berries. They belong to the polyphenol family and are included in the large class of secondary metabolites known as flavonoids, with a core structure in the form of 2-phenylbenzopyrylium or flavylium cation. Anthocyanins mainly derive from six anthocyanidins (aglycone form): cyanidin, delphinidin, pelargonidin, peonidin, petunidin, and malvidin [1,2]. Environmental factors affect anthocyanin production in plants, and it was shown that anthocyanin amount and profiles change with stress conditions. In this Special Issue, the Hinojosa-Gómez et al. [3] study shows that total anthocyanin content in the roselle (*Hibiscus sabdariffa*) calyx increases when a moderate water stress irrigation regime (65% moisture) is applied. It was also observed that extreme water stress (33% moisture) during plant development led to a decrease in anthocyanin content in all the roselle cultivars [3]. This study highlights the importance of the agricultural practices in the anthocyanins content towards an increase in plant health-promoting benefits.

In the last decades, anthocyanins have caught the interest of industry because they are water-soluble pigments present in high amounts in plants, which display interesting nutraceutical and technological properties. Mattioli et al. [1] summarized for this Special Issue the existing procedures to extract, isolate and characterize anthocyanins. The most frequently employed approach to extract anthocyanins from biological matrices consists in the use of a solvent, being water, ethanol, methanol, acetone, and acetonitrile the more widely used. Natural deep eutectic solvents (NADES) are promising green alternatives to conventional solvents to extract anthocyanins from natural products. NADES represent a green chemistry alternative to organic solvents, and have some advantages, including immediately available components, low cost, easy preparation, low toxicity, and high sustainability. The techniques used to extract anthocyanins are non-selective, so after this procedure, it is necessary to purify them. For aqueous extracts, an effective and cheap purification approach is the absorption of anthocyanins on solid phase extraction. In this technique, dissolved anthocyanins are retained in a resin packed column based on their physicochemical features, and then they are separated from other compounds by increasing the polarity with distinct solvents. After extraction and purification, the chromatographic isolation of anthocyanins can be carried out to characterize their structure. Nuclear magnetic resonance analysis is the gold standard for the clear elucidation of chemical structures of novel compounds [1].

This Special Issue provides an overview of the current knowledge on anthocyanins and their health benefits. Particularly, two reviews focus on summarizing results from studies carried out to explore the impact of anthocyanins on cardiovascular and neurodegenerative diseases [1] and type 2 diabetes [2]. In both papers, the authors highlight the value of anthocyanins to counteract the pro-inflammatory state, since this is a major contributing factor in the onset, development, and progression, of those chronic diseases. Regarding neurodegenerative diseases, Mattioli et al. [1] summarize the results of several studies showing that anthocyanins mitigate many of the damaging effects of processes implicated in neurodegeneration such as oxidative and nitrosative stress, glial inflammation, excitotoxicity, protein aggregation, and the induction of apoptotic signaling proteins. About cardiovascular diseases, the same authors [1] show that anthocyanins have protective effects by targeting different pathways that are involved in the pathogenesis of metabolic syndromes leading to cardiovascular disease. The studies reviewed indicate that anthocyanins positively affect the lipid profile by reducing total cholesterol, low-density lipoprotein, cholesterol, and triglycerides levels. Furthermore, anthocyanins increase the ratio of polyunsaturated fatty acids and decrease the amount of saturated fatty acids in plasma. Anthocyanins also have antioxidant proprieties; for example, they increase the activity of the high-density lipoprotein-associated paraoxonase 1, which breaks down harmful oxidized lipids in lipoproteins, in macrophages and in atherosclerotic plaques. Finally, it is also shown that anthocyanins have vasorelaxant, antihypertensive, antihemolytic activities and attenuate platelet aggregation [1]. The inverse relationship between anthocyanins intake and the total cholesterol:high density lipoprotein (HDL) cholesterol levels was also observed in the cross-sectional study carried out by Hershey et al. [4] and published in this issue. Furthermore, the joint effect of a low anthocyanin intake and low physical exercise more than doubled the relative risk of having HDL cholesterol<40 mg/dL, compared to high anthocyanins/high activity joint exposure, although the results lack statistical significance [4].

Diabetes is a prime risk factor for cardiovascular disease, and its global prevalence has increased rapidly over the last few decades. In this Special Issue, the review written by Oliveira et al. [2] explores the anthocyanins’ antidiabetic potential. The literature analysis shows that anthocyanins have therapeutic properties in diabetes through different pathways and mechanisms. Anthocyanins can reduce diabetes-related hyperglycemia and hemoglobin A1c levels. The authors also discuss the different mechanisms found, by which anthocyanins inhibit α-amylase and α-glucosidase, as well as interfere with glucose transport, glycogenolysis and lipid metabolism by different molecular pathways. Anthocyanins also control blood glucose by normalizing insulin secretion and resistance. As mentioned, anthocyanins have anti-inflammatory and antioxidant activities, which are important in the control of the apoptotic factors and consequent pancreatic β cells protection. These antioxidant properties are important to decrease not only the cardiovascular damage but also the renal one [2]. Authors from both review papers alert for the need to perform double-blind clinical trials recruiting a high number of patients to unequivocally evaluate the efficacy of anthocyanins. The importance of carrying out studies to determine and explain mechanisms attributed to each individual anthocyanin is also pointed out.

Anthocyanins could have both chemopreventive and therapeutic effects against various types of cancer. In this Special Issue, four papers explored the anthocyanins’ anti-cancer effects [5,6,7,8]. Two of those papers report the results of in vitro studies carried out, by the same research group, to evaluate the anticancer effects of *Vitis coignetiae Pulliat* [5,6]. The authors demonstrated that anthocyanins extracted from the fruits act as inhibitors of NF-κB (factor nuclear kappa B) in MCF-7 cells. The authors suggest anthocyanins inhibit the tumor necrosis factor-α (TNF-α) effect by suppressing genes involved in cancer cell proliferation, invasion, adhesion, and angiogenesis in a NF-κB dependent manner. Moreover, it was observed that anthocyanins have anti-metastatic effects by suppressing the proliferation, adhesion of cancer cells to human umbilical vein endothelial cells, and invasion, as well as the gene expression involving cell proliferation, invasion, and angiogenesis [5]. In the other study, Paramanantham et al. [6] showed that anthocyanins extracted from *Vitis coignetiae Pulliat* also increase cisplatin sensitivity by inhibiting the protein kinase B (Akt) and NF-κB activity of MCF-7 cells that show relative intrinsic cisplatin resistance. Therefore, it may be speculated that adding anthocyanins to cisplatin could be an alternative therapeutic option by combining TNF-α inhibitor and cisplatin in human breast cancer [6]. Anthocyanins from the fruits of *Vitis coignetiae Pulliat* also have anti-cancer effects in terms of proliferation, migration, and invasion at low concentrations (10–100 µg/mL), on Hep3B human hepatocellular carcinoma cells, as shown in another paper of this issue [7]. As with breast cancer cells, the anthocyanins’ anti-cancer effects in Hep3B h cells are mediated through the inhibition of NF-κB and its target proteins, which are involved in cancer cell proliferation, invasion, and angiogenesis. However, results seem to indicate that when TNF-α is high, the anthocyanins’ anti-cancer effect decreases. This result highlights the need for further research to validate the anti-cancer effects of anthocyanins in highly metastatic cancer or far advanced cancers characterized by high TNF-α levels [7]. Simas Frauches et al. [8] carried out a study which aimed to characterize and compare the constituents of jabuticaba (*Myrciaria jaboticaba*), Jamun berry (*Syzygium cumini*), and Malay apple (*Syzygium malaccense*) extracts and their effect on antioxidant activity in vitro and antiproliferative effects on human colon adenocarcinoma cells. The results, published in this Special Issue, show a decrease in cell viability of HT-29 cells after treatment with Myrtaceae fruits extracts. Moreover, the studied anthocyanin-rich extracts induced G2/M cell cycle arrest and, consequently, apoptosis. Taken together, the results indicate that peel powders from Myrtaceae fruits are an important source of natural antioxidants and could be used to suppress colon cancer cell growth [8].

In conclusion, this Special Issue brings together information about the extraction of anthocyanins from natural sources and their health-promoting properties. In future, it will be important to design appropriate clinical studies to assess the potential benefits of anthocyanins on metabolic disorders, as well as interactions of these flavonoids with the gut microbiome.
